# Two cases of food aversion with semantic dementia

**DOI:** 10.1080/13554794.2016.1149592

**Published:** 2016-03-10

**Authors:** Alexandra E. Thompson, Camilla N. Clark, Christopher J. Hardy, Phillip D. Fletcher, John Greene, Jonathan D. Rohrer, Jason D. Warren

**Affiliations:** ^a^ Dementia Research Centre, UCL Institute of Neurology, University College London, London, UK; ^b^ Medical School, University of Adelaide, Adelaide, Australia; ^c^ Southern General Hospital, Glasgow, UK

**Keywords:** Anorexia, eating behavior, semantic dementia, frontotemporal dementia, bulimia

## Abstract

Accounts of altered eating behavior in semantic dementia generally emphasize gluttony and abnormal food preferences. Here we describe two female patients with no past history of eating disorders who developed early prominent aversion to food in the context of an otherwise typical semantic dementia syndrome. One patient (aged 57) presented features in line with anorexia nervosa while the second patient (aged 58) presented with a syndrome more suggestive of bulimia nervosa. These cases add to the growing spectrum of apparently dichotomous behavior patterns in the frontotemporal dementias and illustrate a potentially under-recognized cause of eating disorders presenting in later life.

## Introduction

Altered food preferences and abnormal eating behaviors are often early and prominent symptoms of the frontotemporal lobar degenerations (FTLD). Eating abnormalities are a defining feature of the behavioral variant of frontotemporal dementia as reflected in current diagnostic criteria for this syndrome (Ahmed et al., 2014; Ikeda, Brown, Holland, Fukuhara, & Hodges, 2001; Mendez, Licht, & Shapira, 2008; Rascovsky et al., 2011; Snowden et al., 2001) but also commonly develop in association with semantic dementia (Ahmed et al., 2014; Ikeda et al., 2001; Rohrer & Warren, 2010; Snowden et al., 2001). Abnormal eating behaviors in these patients typically take the form of gluttony, pathological sweet tooth, binge eating, and loss of table manners. Such behaviors may reflect impaired response inhibition, loss of social understanding, and altered regulation of satiety mechanisms and food-associated reward valuation, all attributable to the disruption of brain mechanisms that normally modulate feeding behavior according to biological and social context (Ismail et al., 2008; Whitwell et al., 2007; Woolley et al., 2007). However, the spectrum of eating abnormalities in FTLD is diverse and includes less well-defined features such as food faddism, consumption of noncanonical food combinations, and mouthing or ingestion of non-food items (Ikeda et al., 2001; Piwnica-Worms, Omar, Hailstone, & Warren, 2010; Snowden et al., 2001). Degraded semantic processing of food and compulsive hyperorality may contribute to eating abnormalities in FTLD, particularly in more advanced disease (Omar, Mahoney, Buckley, & Warren, 2013).

Less well recognized than food-seeking behaviors in FTLD are behaviors that signal aversion to food. Anorexia has frequently been described as a feature of Alzheimer’s disease and indeed, commonly accompanies healthy ageing; reduced food intake in these populations may reflect a multifactorial interaction of social, behavioral, and cognitive factors, often exacerbated by medication effects and comorbidities and potentially underpinned by more specific metabolic derangements (Donini et al., 2013; Fadel, Jolivalt, & Reagan, 2013; Luca, Luca, & Calandra, 2015; Morley, 2015; Robles et al., 2009; Roy, Shatenstein, Gaudreau, Morais, & Payette, 2015; Sergi, De Rui, Coin, Inelmen, & Manzato, 2013; Snowden et al., 2001; Thomas, 2009). In contrast, food aversion in FTLD runs clearly counter to the prevailing tendency of these patients to exhibit gluttony and food craving. While symptoms suggesting food aversion have been listed in a variable minority of cases in larger published series of patients with FTLD (Ikeda et al., 2001; Snowden et al., 2001), these symptoms have not been characterized in detail and remain poorly understood.

Here we describe two patients seen in our specialist cognitive clinic at the National Hospital for Neurology and Neurosurgery, London, with semantic dementia fulfilling current diagnostic criteria (Gorno-Tempini et al., 2011) who exhibited forms of food aversion as a significant clinical issue. Both patients gave informed consent to participate, in line with Declaration of Helsinki guidelines.

## Case 1

This 57-year-old right-handed retired social worker presented with a 7-year history of progressive word finding difficulty, poor concentration, and emotional lability. Her speech had become circumlocutory and tangential and she had difficulty understanding others. More recently she had had difficulty recognizing faces of acquaintances. She developed an intense liking for a small repertoire of popular songs but found sirens and similar environmental noises unpleasant and distressing. She also complained of fatigue and other ill-defined somatic sensations. Her past medical history included childhood polio, scoliosis, hypothyroidism, and migraine; there was no previous suggestion of an eating disorder. Her family history was censored as she was adopted.

Neuropsychological assessment (see Table 1) demonstrated marked impairments of naming and single word comprehension and in addition, impaired memory for faces. The general neurological examination revealed features in keeping with previous poliomyelitis, but was otherwise unremarkable.

**Table 1.** Summary of neuropsychological findings in patients with semantic dementia and food aversion.

COGNITIVE DOMAIN	CASE 1	CASE 2
General characteristics		
Age	57	58
Symptom duration (years)	7	5
MMSE (/30)	25	24
General intellect		
WASI Verbal IQ	**76**	**66**
WASI Performance IQ	107	99
Executive skills		
Stroop D-KEFS color naming (s)	37	**53**
Stroop D-KEFS word naming (s)	22	25
Stroop D-KEFS ink color naming (s)	40	84
Trails Part A (s)	19	40
Trails Part B (s)	70	122
Language skills		
GNT (/30)	**2**	**0**
BPVS (/150)	117*	52*
Concrete synonyms (/25)^†^	**18**	**12**
Abstract synonyms (/25)^†^	**12**	**13**
Episodic memory		
RMT faces (/50)	**28**	42
RMT words (/50)	39	43
Other skills		
WMS-R Digit Span Forward (max)	8	6
WMS-R Digit Span Reverse (max)	7	4
GDA (/24)	4	**2**
VOSP Object Decision (/20)	14	16

Raw scores are shown with maximum scores in parentheses for each neuropsychological test unless otherwise indicated. Scores at or below the 5th percentile on standardized tests based on published norms are indicated in **bold**. * Below 5th percentile referenced to a local cohort of 15 healthy age-matched females. ^†^Warrington, McKenna, and Orpwood (1998); BPVS, British Picture Vocabulary Scale (Dunn, Dunn, & Whetton, 1982); D-KEFS, Delis Kaplan Executive Function System (Delis, Kaplan, & Kramer, 2001); GDA, Graded Difficulty Arithmetic (Jackson & Warrington, 1986); GNT, Graded Naming Test (McKenna & Warrington, 1983); MMSE, Mini-Mental State Examination score (Folstein, Folstein, & McHugh, 1975); NART, National Adult Reading Test (Nelson, 1982); PAL, Paired Associate Learning; RMT, Recognition Memory Test (Warrington, 1984); VOSP, Visual Object and Spatial Perception Battery (Warrington & James, 1991); WASI, Wechsler Abbreviated Scale of Intelligence (Wechsler, 1999); WMS-R, Wechsler Memory Scale Revised (Wechsler, 1987).

**Figure 1.**
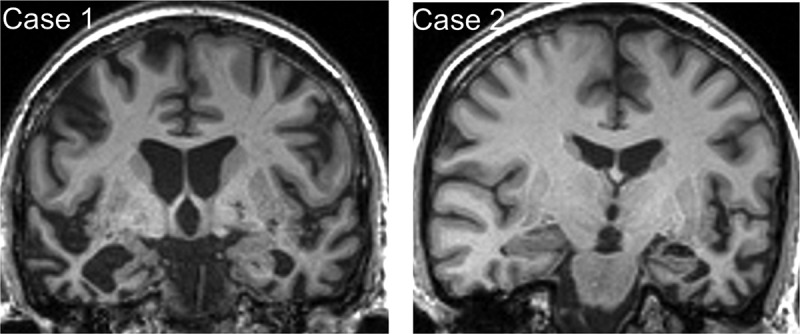
Brain MRI profiles in patients with semantic dementia and food aversion. Representative coronal T1-weighted MR sections through the anterior temporal lobes are presented for each of the patients described; the left hemisphere is shown on the right for both sections. In each case, there is relatively focal, asymmetric atrophy of the anterior temporal lobes, most marked medially and inferiorly (predominantly right-sided though bilateral in Case 1, predominantly left-sided in Case 2).

Brain MRI (Figure 1) showed bilateral though asymmetric anterior, medial, and inferior temporal lobe atrophy, more marked in the right cerebral hemisphere. CSF examination showed levels of neurodegeneration marker proteins within the normal ranges for the local laboratory (total tau 321 pg/mL, beta-amyloid_1-42_ 957 pg/mL, ratio tau:amyloid 0.34), making a diagnosis of underlying Alzheimer’s disease pathology unlikely.

### Eating behavior

Though she had always been regarded by her family as fastidious in her culinary habits there was no history of an eating disorder in her earlier life. Around 10 years previously, she had begun to complain of nausea and anorexia for which no cause was identified. Her food intake diminished and her weight decreased from around nine to approximately seven stone over a period of months. She became obsessed that she was overweight and that her abdominal girth was increasing and her family were unable to dissuade her of this. To their concern and perplexity, she spontaneously placed herself on a rigid and restrictive diet consisting almost exclusively of soy milk, raspberries, and porridge. She did not develop any predilection for sweets or carbohydrates. However, having been a lifelong teetotaller, she began consuming alcohol in modest quantities. Though her weight stabilized, this unusual dietary pattern continued over several years prior to her presenting to our service.

## Case 2

This 58-year-old right-handed retired hairdresser presented with a 5-year history of progressive deterioration of language skills, initially noted to affect spelling followed by impaired comprehension of speech and reading material. She had asked family members the meaning of words such as “cholesterol” and “epilepsy” while attempting to read the newspaper. For around 2 years she had had increasing difficulty recognizing faces of acquaintances and celebrities. She was forced to stop work as she was not able to carry out clients’ instructions reliably, though she remained able to use credit cards and managed her own grocery shopping. She became less empathic toward her family, more outspoken toward strangers, made extravagant purchases, and exhibited markedly heightened interest in all kinds of music. She had been diagnosed with Raynaud’s phenomenon but had no other medical history of note nor any known family history of dementia or psychiatric illness.

Neuropsychological assessment (Table 1) confirmed severe impairments of naming and single word comprehension. The general neurological examination was unremarkable.

Brain MRI (Figure 1) showed focal asymmetric anterior, medial, and inferior temporal lobe atrophy, more marked in the left cerebral hemisphere with “knife-blade” atrophy of the left temporal pole.

### Eating behavior

Her family reported that she had always been interested in “healthy eating”, but identified a clear change in her eating behavior, in that she had become obsessed with her weight and body image, frequently complaining that she was overweight (in fact, she retained her lifelong slim build). This was regarded by her family as highly uncharacteristic and a source of considerable concern; however, the behavior had intensified over several years. She became increasingly rigid when planning meals, stringently avoiding foods she considered high in calories in favor of a narrow repertoire of low-fat supermarket ready meals. She would become distressed if encouraged to broaden this repertoire. She talked continually about food and meals and frequently bought her children gifts of sweet foods. At the same time, she began periodically to binge on sweets, often attempting to hide this from others, and would seek out sweets that other family members had hidden from her. She began regularly to use a personal “fat-vibrating” machine, around four times a day and particularly after episodes of bingeing. This behavior led to difficulties at work, where she had been found secluded in the toilet bingeing on sweets. Attempted interventions by her family and a trial of fluoxetine were unsuccessful in controlling the behaviors.

## Discussion

These patients developed various symptoms of aversion to food in the context of an otherwise typical syndrome of semantic dementia. Comparing their symptoms against current DSM-5 criteria for eating disorders (American Psychiatric Association, 2013), Case 1 exhibited features in line with anorexia nervosa: notably, reduced energy intake leading to significant weight loss, fear or intense dislike of gaining weight and preoccupation with weight and fixed false beliefs about body habitus despite repeated reassurance and encouragements to eat from family members. Case 2 exhibited certain features more in line with bulimia nervosa: besides a pervasive preoccupation with low calorie diets and body habitus, she seemed compelled to engage in discrete and often clandestine food binges followed by compensatory compulsive and excessive exercising as a sustained behavior pattern. These cases add to the already diverse spectrum of behavioral alterations described in association with semantic dementia and other syndromes of FTLD. The development of food aversion in these patients is of particular interest as a window on the pathophysiology of eating disorders in later life and especially in the context of neurodegenerative disease. In addition, when set against the more widely described profile of gluttony and hyperphagia, such cases illustrate an apparent dichotomization of FTLD-associated eating behaviors that suggests a parallel with other behavioral dichotomies previously described in these patients, in the realms of affective, sexual, and homeostatic reactivity (Clark & Warren, 2015).

Detailed analyses of eating behaviors in patients with FTLD suggest that aversion to food is not uncommon. Despite this, food aversion has received little attention in the extensive literature devoted to eating abnormalities in semantic dementia and FTLD. In one series (Ikeda et al., 2001), decreased appetite was reported in 8% of patients and a sensation of being “overfull” in 16% of patients with semantic dementia; these features contrasted with increased appetite, food seeking and binge eating, reported in around 30–40% of patients with semantic dementia. Some patients with semantic dementia apparently ate less than healthy individuals when monitored in a free feeding study under controlled laboratory conditions (Woolley et al., 2007) and these patients report less hunger than patients with behavioral variant frontotemporal dementia (Ahmed et al., 2014). Binge eating might constitute a phenomenological bridge between hyperphagia and gluttony and eating disorders such as bulimia, characterized by binge—rejection cycling; moreover, selectivity of oral intake or food faddism is common in semantic dementia, occurring in around 60–70% of cases (Ikeda et al., 2001; Snowden et al., 2001). Taken together, this evidence suggests that food aversion lies on a continuum with the hyperphagia and disordered food preferences more commonly reported in semantic dementia. In support of this interpretation, there is no simple relation between eating behaviors and physiological and neuro-hormonal markers of food intake and satiety in FTLD (Ahmed et al., 2014; Woolley et al., 2014).

Although direct neuroanatomical correlation was not possible for the present cases, focal anterior temporal lobe atrophy is a plausible candidate substrate for the spectrum of eating abnormalities these patients exhibit. Binge eating, hyperphagia, and pathological sweet tooth have previously been linked to disintegration of a fronto-insular, anterior temporal and mesolimbic neural network in FTLD (Whitwell et al., 2007; Woolley et al., 2007): Within this network, anterior temporal cortex may play a critical role in linking the sensory perception of food and homeostatic signals with semantic associations and social context and the programming of appropriate behaviors (Omar et al., 2013). Temporal lobe regions have also been implicated in the pathophysiology of primary eating disorders of younger life (Van den Eynde et al., 2012; Zhu et al., 2012), albeit in the context of widely distributed structural and functional neuroanatomical alterations. Beyond the domain of eating per se, there may be additional similarities between these primary eating disorders of younger life and FTLD: for example, abnormalities of emotional and social cognition, hallmarks of both behavioral variant frontotemporal dementia and semantic dementia, are increasingly recognized in association with anorexia nervosa (Fonville, Giampietro, Surguladze, Williams, & Tchanturia, 2013; McAdams & Krawczyk, 2011), arguing for shared pathophysiological mechanisms. It has been proposed that eroded neural templates for complex behavioral routines such as feeding might potentially allow emergence of behaviors with apparently “opposite” valence following disintegration of a common brain network architecture in FTLD (Clark & Warren, 2015); however, this remains to be substantiated. It is unclear to what extent these changes in food intake in semantic dementia reflect an inability to attribute biological and social meaning to external food-related and internal satiety signals.

The burden of anorexia in older populations is difficult to assess accurately but it is likely to be substantial and to have been underestimated (Donini et al., 2013; Luca et al., 2015; Roy et al., 2015; Thomas, 2009). Feeding abnormalities in older people comprise a spectrum ranging from rare cases of fully fledged eating disorders fulfilling DSM-5 criteria (American Psychiatric Association, 2013; Luca et al., 2015) to disordered regulation of satiety and dissatisfaction with body weight, which are probably much more common (Donini et al., 2013; Roy et al., 2015; Thomas, 2009). The most important neurodegenerative disease contributing to this spectrum is Alzheimer’s disease, in which anorexia, weight loss, and inanition are often significant clinical issues (Ismail et al., 2008; Sergi et al., 2013). These features have a complex causation that may reflect the interaction of a “hypermetabolic” neurohormonal state that tends to promote cachexia with cognitive, affective, behavioral, and social factors that tend to limit diet and food intake (Donini et al., 2013; Fadel et al., 2013; Luca et al., 2015; Morley, 2015; Roy et al., 2015; Sergi et al., 2013; Thomas, 2009). Though amplified in Alzheimer’s disease, similar factors are likely to operate more widely in the older population and it remains unclear to what extent anorexia of later life might be underpinned by other, unrecognized neurodegenerative conditions such as those in the FTLD spectrum.

Based on the case histories presented here, we argue that diseases in the FTLD spectrum should be considered in older people with anorexia and other eating disorders, particularly where accompanied by prominent cognitive, behavioral, or social dysfunction. From a neurobiological perspective, we hope these cases will motivate further systematic analyses of the eating behavior profile of FTLD in larger patient cohorts, ideally with longitudinal assessments and structural and functional neuroimaging, metabolic and neurohormonal correlation. From a broader clinical perspective, environmental modification strategies and medications that modulate central reward and satiety circuitry are potential avenues to manage eating disorders in the setting of neurodegenerative disease (McDaniel, Hunt, Hackes, & Pope, 2001; Singam, Walterfang, Mocellin, Evans, & Velakoulis, 2013): If patients are to benefit from such approaches, the first steps are to suspect an eating disorder, to enquire with the caregiver about eating abnormalities that may not be volunteered, and to assess nutritional state and feeding as part of the comprehensive evaluation of patients with dementia.
